# P-321. Comparative Acceptability of HIV Pre-Exposure Prophylaxis Regimens

**DOI:** 10.1093/ofid/ofaf695.540

**Published:** 2026-01-11

**Authors:** Max Shteiman, Gerald D Denton, Connor M Solan

**Affiliations:** The University of Queensland–Ochsner Clinical School, New Orleans, LA; The University of Queensland–Ochsner Clinical School, New Orleans, LA; University of Minnesota, Cottage Grove, Minnesota

## Abstract

**Background:**

Pre-Exposure Prophylaxis (PrEP) for Human Immunodeficiency Virus (HIV) should be recommended for sexually active men who have sex with men (MSM). PrEP may be administered in multiple ways. Given their differences in routes of administration, scheduling, and potential adverse effects, patients may have preferences for their form of PrEP. However, existing literature has not yet compared patient acceptability of the newest available option, intramuscular (IM) cabotegravir (CAB), with more established alternatives: emtricitabine-tenofovir disoproxil fumarate (TDF-FTC) and emtricitabine-tenofovir alafenamide (TAF-FTC). The aim of this study was to identify patient acceptability of these PrEP options, as well as understand the factors that may affect preferences for daily or event-based administration regimens.

**Methods:**

Using a cross-sectional design, we administered a survey tool to 340 eligible patients already taking PrEP at a large regional medical center in New Orleans, Louisiana. The survey collected data on patient demographics, PrEP and sexual practices, and HIV risk, which was subsequently analyzed using chi-squared testing.

**Results:**

The analysis of 141 completed surveys (41.5%) revealed that patients overwhelmingly self-identified as male (97%), White (89%), and gay (87%), and represented a wide range of age groups. IM CAB was the preferred PrEP option (p< 0.05) among participants who reported having one to five sexual partners in the past three months and having intercourse on a monthly or weekly basis.

**Conclusion:**

This study identified a statistically significant preference for IM CAB among participants with specific self-reported ranges of sexual partners and frequency of intercourse. This finding contributes to a growing set of research on PrEP options and highlights a difference in acceptability of various pharmacological options, which may guide provider interactions and considerations when discussing PrEP with patients. To improve the power of this study, the survey tool should be distributed to a more racially and sexually diverse group of participants with free-text options for qualitative analysis.

**Disclosures:**

All Authors: No reported disclosures

